# Supplementary material for: Development of a computer-aided design software for the quantitative evaluation of aesthetic damage
Source: PLoS One. 2019 Dec 18;14(12):e0226322. doi: 10.1371/journal.pone.0226322 (PMC6919621; doi:10.1371/journal.pone.0226322)
Supplement: S1 Code site — 1. (ZIP) [file pone.0226322.s001.zip › [EESC_jr][Projeto]Relatorio_07_21/reports/2/2017.07.11/07:08:26/report.pdf]

Preâmbulo

Na data de 11/07/2017 no horário , o(a) Perito(a) Professor Doutor Nelson Sakaguti, Cirurgião Dentista, designado(a)para proceder ao exame pericial solicitado em: fabicho macho, do sexo Feminino, nascido(a) em 21/02/1980, ocupação pintora, portador(a) do RG 987 ssp, cujo endereço reis alves 23, passa a expor a avaliação de dano estético corporal.

Exame objetivo da lesão

A lesão ora objeto de análise teve como natureza ocorrido em 01/01/1970. O prejuízo estético consiste em lesão cicatricial consolidada na região: Ombros, Nádegas, Púbis no lado superior, direito.

Trata-se de cicatriz Hipertrófica de tamanho/frequência Única: Retilínea de orientação Horizontal de pigmentação Normocrômica medindo 10 centímetro(s) na horizontal, 3 centímetros na vertical e 2 milímetros de Profundidade. De contornos Regulares. A lesão tem repercussão é visível a 50 centímetros nas relações íntimas. A lesão é visível a 3 (três) metros nas relações sociais. A lesão tem repercussão na vida diária familiar, social, desportiva conforme fotos anteriores e atuais..

Conclui-se que classificada segundo os parâmetros de dano estético AIPE - Análise do Impacto do Prejuízo Estético (escala de seis níveis e pontuação entre 0 e 50 pontos), como sendo dano estético relativo à área 1, a lesão é caracterizada por 3 pontos e grau de prejuízo estético: Leve.

Escore final: 3;
Grau de prejuízo: Leve.

fousp, 11/07/2017

____

Professor Doutor Nelson Sakaguti
